# Supplementary figures and images for: Use and effectiveness of dapagliflozin in patients with type 2 diabetes mellitus: a multicenter retrospective study in Taiwan
Source: PeerJ. 2020 Nov 17;8:e9998. doi: 10.7717/peerj.9998 (PMC7678460; doi:10.7717/peerj.9998)

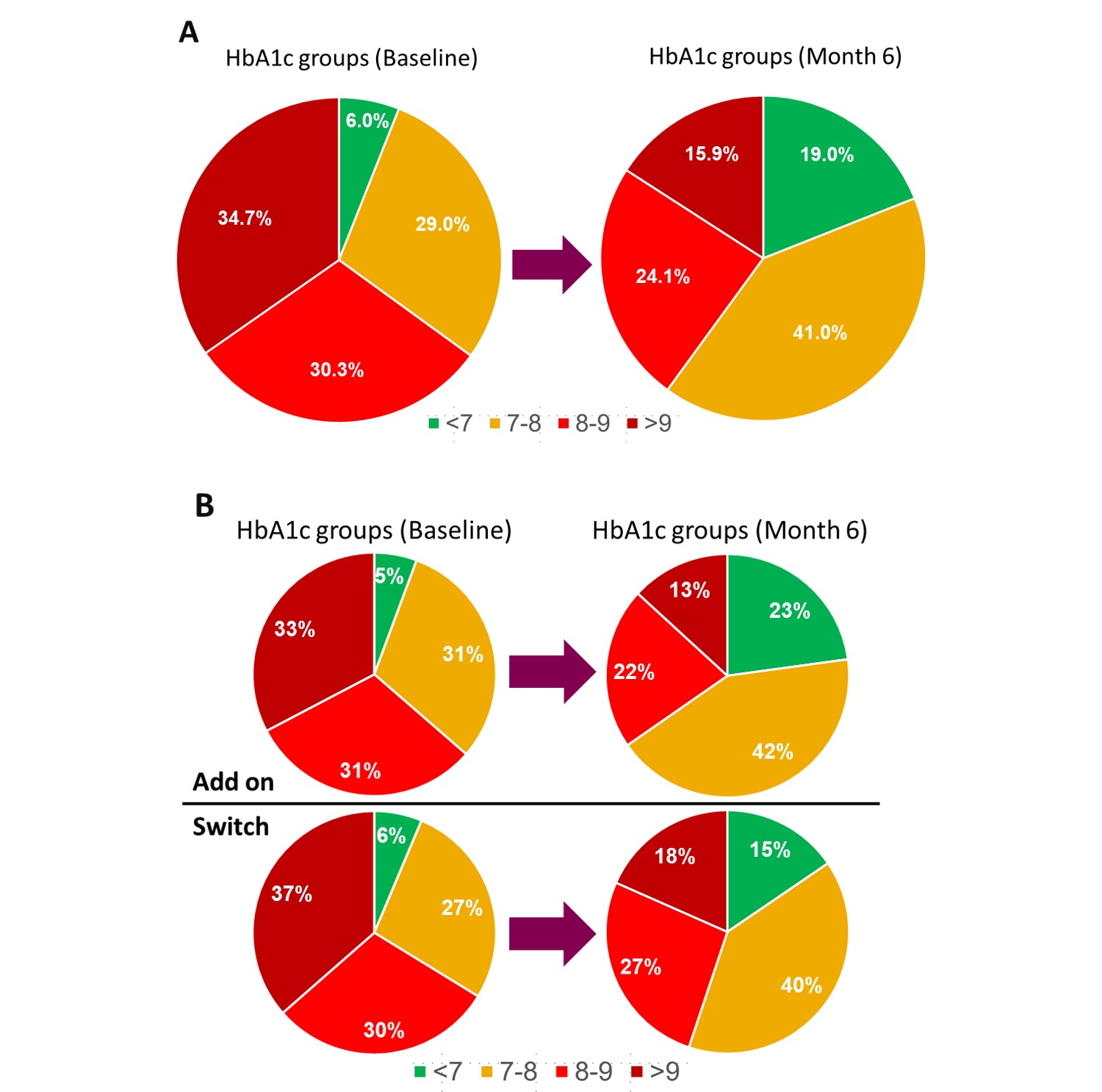

Supplement: Supplemental Information 1 [file peerj-08-9998-s001.jpg]

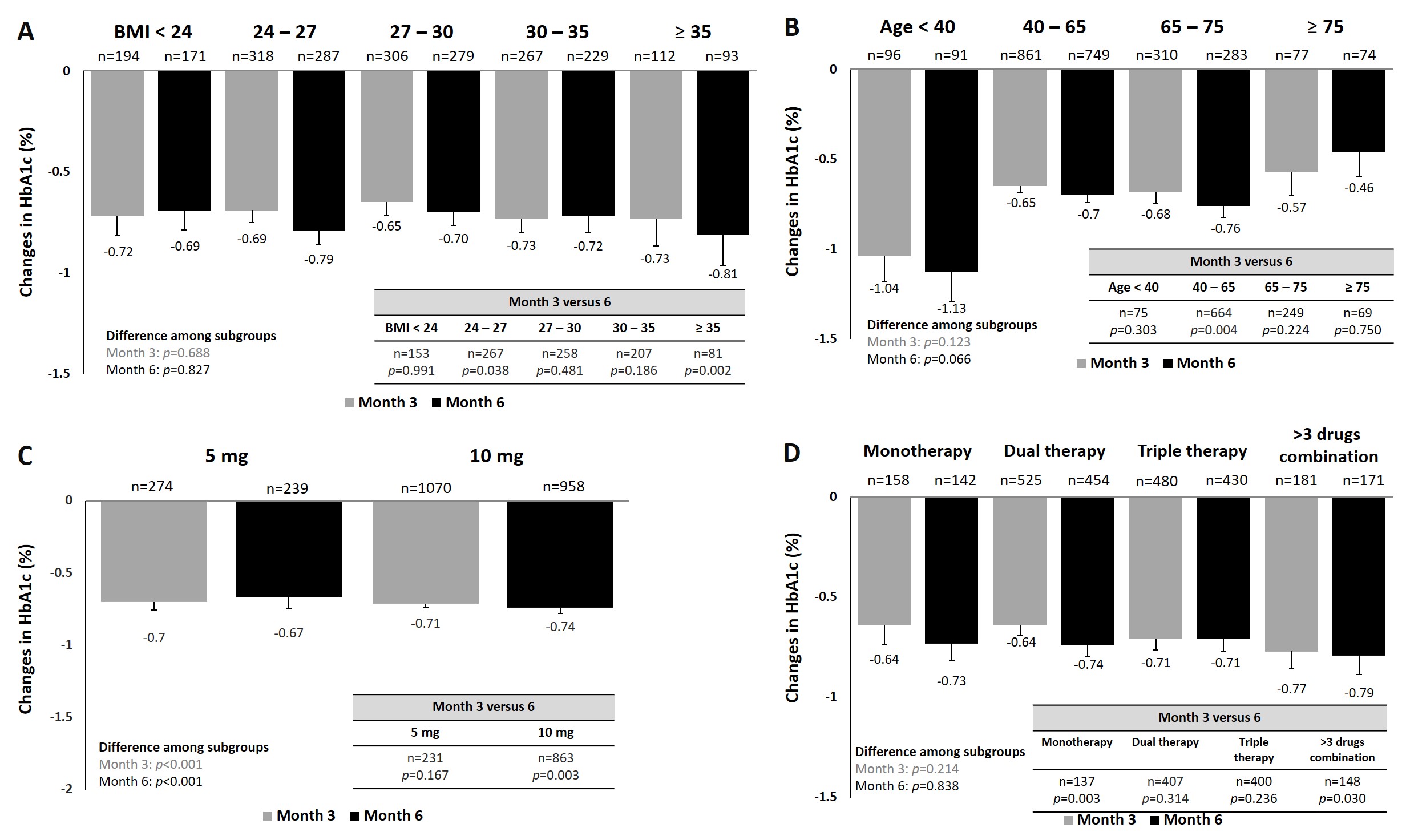

Supplement: Supplemental Information 2 [file peerj-08-9998-s002.jpg]

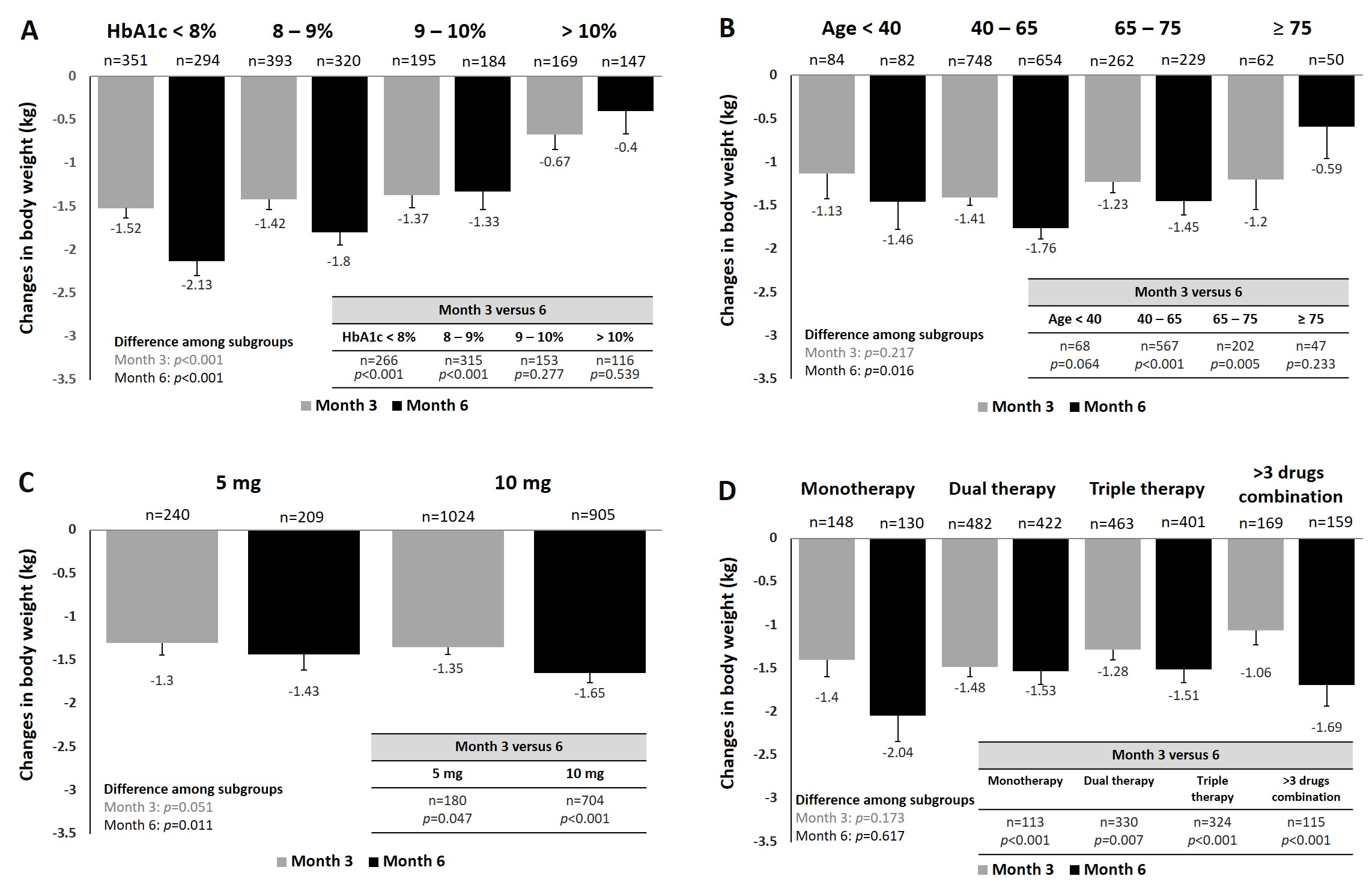

Supplement: Supplemental Information 3 [file peerj-08-9998-s003.jpg]
