# Supplementary material for: Use and effectiveness of dapagliflozin in patients with type 2 diabetes mellitus: a multicenter retrospective study in Taiwan
Source: PeerJ. 2020 Nov 17;8:e9998. doi: 10.7717/peerj.9998 (PMC7678460; doi:10.7717/peerj.9998)
Supplement: Supplemental Information 4 [file peerj-08-9998-s004.zip › Clinical dataset/[D1690R00022] Codebook.pdf]

Datasets For Study D1690R00022		
**Data Set**	**Description of Dataset**	**Location**
CM1	STUDY INDICATION MEDICATIONS CRF	\\Clinical Data Sets\XPT Files\cm1.xpt
CM1_GP	STUDY INDICATION MEDICATIONS CRF	\\Clinical Data Sets\XPT Files\cm1_gp.xpt
CM2	STUDY RELEVANT MEDICATIONS CRF	\\Clinical Data Sets\XPT Files\cm2.xpt
CM2_GP	STUDY RELEVANT MEDICATIONS CRF	\\Clinical Data Sets\XPT Files\cm2_gp.xpt
CM_GP	STUDY INDICATION MEDICATIONS CRF	\\Clinical Data Sets\XPT Files\cm_gp.xpt
DAP	DAPAGLIFLOZIN CRF	\\Clinical Data Sets\XPT Files\dap.xpt
DM	DEMOGRAPHICS CRF	\\Clinical Data Sets\XPT Files\dm.xpt
IE	ELIGIBILITY CRITERIA CRF	\\Clinical Data Sets\XPT Files\ie.xpt
LB	LABORATORY EVALUATION CRF	\\Clinical Data Sets\XPT Files\lb.xpt
MH	MEDICAL HISTORY CRF	\\Clinical Data Sets\XPT Files\mh.xpt
PD	PROTOCOL DEVIATION LIST	\\Clinical Data Sets\XPT Files\pd.xpt
PE	PHYSICAL EXAMINATION CRF	\\Clinical Data Sets\XPT Files\pe.xpt

Study D1690R00022 - STUDY INDICATION MEDICATIONS CRF (cm1)			
**Variable**	**Label**	**Type**	**Source**
STUDYID	Study Identifier	Char	All CRF
DOMAIN	Domain Abbreviation	Char	All CRF
PTNO	Subject No.	Char	All CRF
VISIT	Visit	Char	All CRF
CMTRTYN1	Metformin None	Char	STUDY INDICATION MEDICATIONS CRF
CMTRTVT1	Metformin	Char	STUDY INDICATION MEDICATIONS CRF
CMTRTYN2	Sulfonylurea None	Char	STUDY INDICATION MEDICATIONS CRF
CMTRTVT2	Sulfonylurea	Char	STUDY INDICATION MEDICATIONS CRF
CMTRTYN3	DPP4 inhibitor None	Char	STUDY INDICATION MEDICATIONS CRF
CMTRTVT3	DPP4 inhibitor	Char	STUDY INDICATION MEDICATIONS CRF
CMTRTYN4	Meglitinide None	Char	STUDY INDICATION MEDICATIONS CRF
CMTRTVT4	Meglitinide	Char	STUDY INDICATION MEDICATIONS CRF
CMTRTYN5	Alpha-glucosidase inhibitor None	Char	STUDY INDICATION MEDICATIONS CRF
CMTRTVT5	Alpha-glucosidase inhibitor	Char	STUDY INDICATION MEDICATIONS CRF

Study D1690R00022 - STUDY INDICATION MEDICATIONS CRF (cm1)			
**Variable**	**Label**	**Type**	**Source**
CMTRTYN6	Thiazolidinedione None	Char	STUDY INDICATION MEDICATIONS CRF
CMTRTVT6	Thiazolidinedione	Char	STUDY INDICATION MEDICATIONS CRF
CMTRTYN7	GLP-1 RA None	Char	STUDY INDICATION MEDICATIONS CRF
CMTRTVT7	GLP-1 RA	Char	STUDY INDICATION MEDICATIONS CRF
CMTRTYN8	Insulin None	Char	STUDY INDICATION MEDICATIONS CRF
CMTRTVT8	Insulin	Char	STUDY INDICATION MEDICATIONS CRF
CMINDC	Antihyperglycemic therapy (including injection therapy) at baseline (except Dapagliflozin)	Char	STUDY INDICATION MEDICATIONS CRF

Study D1690R00022 - STUDY INDICATION MEDICATIONS CRF (cm1_gp)			
**Variable**	**Label**	**Type**	**Source**
STUDYID	Study Identifier	Char	All CRF
DOMAIN	Domain Abbreviation	Char	All CRF
PTNO	Subject No.	Char	All CRF
VISIT	Visit	Char	All CRF
CMTRTOTH	Others, specify	Char	STUDY INDICATION MEDICATIONS CRF
CMTRTVT9	Others	Char	STUDY INDICATION MEDICATIONS CRF

Study D1690R00022 - STUDY RELEVANT MEDICATIONS CRF (cm2)			
**Variable**	**Label**	**Type**	**Source**
STUDYID	Study Identifier	Char	All CRF
DOMAIN	Domain Abbreviation	Char	All CRF
PTNO	Subject No.	Char	All CRF
VISIT	Visit	Char	All CRF
CMRELYN1	ACEI or ARB None	Char	STUDY RELEVANT MEDICATIONS CRF
CMRELVT1	ACEI or ARB	Char	STUDY RELEVANT MEDICATIONS CRF
CMRELYN2	Beta blocker None	Char	STUDY RELEVANT MEDICATIONS CRF
CMRELVT2	Beta blocker	Char	STUDY RELEVANT MEDICATIONS CRF
CMRELYN3	Calcium channel blocker None	Char	STUDY RELEVANT MEDICATIONS CRF
CMRELVT3	Calcium channel blocker	Char	STUDY RELEVANT MEDICATIONS CRF
CMRELYN4	Alpha adrenergic antagonist None	Char	STUDY RELEVANT MEDICATIONS CRF
CMRELVT4	Alpha adrenergic antagonist	Char	STUDY RELEVANT MEDICATIONS CRF
CMRELYN5	Diuretic None	Char	STUDY RELEVANT MEDICATIONS CRF
CMRELVT5	Diuretic	Char	STUDY RELEVANT MEDICATIONS CRF

Study D1690R00022 - STUDY RELEVANT MEDICATIONS CRF (cm2)			
**Variable**	**Label**	**Type**	**Source**
CMRELYN6	Statin None	Char	STUDY RELEVANT MEDICATIONS CRF
CMRELVT6	Statin	Char	STUDY RELEVANT MEDICATIONS CRF
CMRELYN7	Fibrate None	Char	STUDY RELEVANT MEDICATIONS CRF
CMRELVT7	Fibrate	Char	STUDY RELEVANT MEDICATIONS CRF
CMRELYN8	Niacin None	Char	STUDY RELEVANT MEDICATIONS CRF
CMRELVT8	Niacin	Char	STUDY RELEVANT MEDICATIONS CRF
CMRELYN9	Ezetimibe None	Char	STUDY RELEVANT MEDICATIONS CRF
CMRELVT9	Ezetimibe	Char	STUDY RELEVANT MEDICATIONS CRF
CMRLYN10	Bile acid sequestrants None	Char	STUDY RELEVANT MEDICATIONS CRF
CMRLVT10	Bile acid sequestrants	Char	STUDY RELEVANT MEDICATIONS CRF
CMRLYN11	Anti-platelet drug None	Char	STUDY RELEVANT MEDICATIONS CRF
CMRLVT11	Anti-platelet drug	Char	STUDY RELEVANT MEDICATIONS CRF

Study D1690R00022 - STUDY RELEVANT MEDICATIONS CRF (cm2)			
**Variable**	**Label**	**Type**	**Source**
CMRLYN12	Anti-coagulant drug None	Char	STUDY RELEVANT MEDICATIONS CRF
CMRLVT12	Anti-coagulant drug	Char	STUDY RELEVANT MEDICATIONS CRF
CMRELYN	Is there any cardiovascular treatment, anti-hypertension or anti-lipid medication use from 12 months prior to baseline to Month 6?	Char	STUDY RELEVANT MEDICATIONS CRF

Study D1690R00022 - STUDY RELEVANT MEDICATIONS CRF (cm2_gp)			
**Variable**	**Label**	**Type**	**Source**
STUDYID	Study Identifier	Char	All CRF
DOMAIN	Domain Abbreviation	Char	All CRF
PTNO	Subject No.	Char	All CRF
VISIT	Visit	Char	All CRF
CMRELOTH	Others, specify	Char	STUDY RELEVANT MEDICATIONS CRF
CMRLVT13	Others	Char	STUDY RELEVANT MEDICATIONS CRF

Study D1690R00022 - STUDY INDICATION MEDICATIONS CRF (cm_gp)			
**Variable**	**Label**	**Type**	**Source**
STUDYID	Study Identifier	Char	All CRF
DOMAIN	Domain Abbreviation	Char	All CRF
PTNO	Subject No.	Char	All CRF
VISIT	Visit	Char	All CRF
CMDOSE	Dose	Char	STUDY INDICATION MEDICATIONS CRF
CMONGO	Continue Treatment	Char	STUDY INDICATION MEDICATIONS CRF
CMSTDAT1	Start Date	Num	STUDY INDICATION MEDICATIONS CRF
CMENDAT1	End Date	Num	STUDY INDICATION MEDICATIONS CRF
CMDOSFRQ	Freq.	Char	STUDY INDICATION MEDICATIONS CRF

Study D1690R00022 - DAPAGLIFLOZIN CRF (dap)			
**Variable**	**Label**	**Type**	**Source**
STUDYID	Study Identifier	Char	All CRF
DOMAIN	Domain Abbreviation	Char	All CRF
PTNO	Subject No.	Char	All CRF
VISIT	Visit	Char	All CRF
REASON1	What is the reason of the subject changing current regimen?	Char	DAPAGLIFLOZIN CRF
INTCO	Intolerance of current regimen, please specify	Char	DAPAGLIFLOZIN CRF
POORCO	Poor compliance to current regimen, please specify	Char	DAPAGLIFLOZIN CRF
OTHCO1	Others, please specify	Char	DAPAGLIFLOZIN CRF
OTHCO2	Others, please specify	Char	DAPAGLIFLOZIN CRF
DAPDAT1	Date of Initiation of dapagliflozin	Num	DAPAGLIFLOZIN CRF
REASON2	What is the reason of the subject choosing dapagliflozin therapy than other anti-diabetic medications?	Char	DAPAGLIFLOZIN CRF
CHANGEYN	Did the subject change current regimen?	Char	DAPAGLIFLOZIN CRF

Study D1690R00022 - DEMOGRAPHICS CRF (dm)			
**Variable**	**Label**	**Type**	**Source**
STUDYID	Study Identifier	Char	All CRF
DOMAIN	Domain Abbreviation	Char	All CRF
PTNO	Subject No.	Char	All CRF
VISIT	Visit	Char	All CRF
VISDAT	Date of Visit	Num	DEMOGRAPHICS CRF
RFICDTC	Date of Informed Consent Signed	Num	DEMOGRAPHICS CRF
BRTHDAT	Date of Birth	Num	DEMOGRAPHICS CRF
HEIGHT	Height	Num	DEMOGRAPHICS CRF
SEX	Gender	Char	DEMOGRAPHICS CRF

Study D1690R00022 - ELIGIBILITY CRITERIA CRF (ie)			
**Variable**	**Label**	**Type**	**Source**
STUDYID	Study Identifier	Char	All CRF
DOMAIN	Domain Abbreviation	Char	All CRF
PTNO	Subject No.	Char	All CRF
VISIT	Visit	Char	All CRF
EX01	1. Outpatient equal to or more than 20 years of age.	Char	ELIGIBILITY CRITERIA CRF
EX02	2. T2DM patient initiated dapagliflozin after May 1st 2016 as a second line or third line oral anti-diabetic therapy, either as add-on or switching from one to another. Or initiating dapagliflozin as adjunctive therapy for T2DM subjects treated with insulin.	Char	ELIGIBILITY CRITERIA CRF
EX03	3. Completed follow-up of at least 6 months regardless of continuation on dapagliflozin therapy.	Char	ELIGIBILITY CRITERIA CRF
IN01	4. Will provide completed and signed written informed consents.	Char	ELIGIBILITY CRITERIA CRF
IN02	1. Subjects with a history of SGLT2 inhibitor therapy other than dapagliflozin prior to Baseline.	Char	ELIGIBILITY CRITERIA CRF
IN03	2. Subjects with Type 1 diabetes.	Char	ELIGIBILITY CRITERIA CRF
IN04	3. Treatment with other investigational drugs concurrently during the retrospective data collection period.	Char	ELIGIBILITY CRITERIA CRF

Study D1690R00022 - LABORATORY EVALUATION CRF (lb)			
**Variable**	**Label**	**Type**	**Source**
STUDYID	Study Identifier	Char	All CRF
DOMAIN	Domain Abbreviation	Char	All CRF
PTNO	Subject No.	Char	All CRF
VISIT	Visit	Char	All CRF
LBDAT	Date of Sample Collection	Num	LABORATORY EVALUATION CRF
LBORRES1	Glycated Haemoglobin (HbA1c) Value	Char	LABORATORY EVALUATION CRF
LBSTAT1	Glycated Haemoglobin (HbA1c) ND	Char	LABORATORY EVALUATION CRF
LBORRES2	Fasting Plasma Glucose (FPG) Value	Char	LABORATORY EVALUATION CRF
LBSTAT2	Fasting Plasma Glucose (FPG) ND	Char	LABORATORY EVALUATION CRF
LBORRES3	Total Cholesterol Value	Char	LABORATORY EVALUATION CRF
LBSTAT3	Total Cholesterol ND	Char	LABORATORY EVALUATION CRF
LBORRES4	High Density Lipoprotein (HDL) Value	Char	LABORATORY EVALUATION CRF
LBSTAT4	High Density Lipoprotein (HDL) ND	Char	LABORATORY EVALUATION CRF
LBORRES5	Low Density Lipoprotein (LDL) Value	Char	LABORATORY EVALUATION CRF
LBSTAT5	Low Density Lipoprotein (LDL) ND	Char	LABORATORY EVALUATION CRF
LBORRES6	Triglycerides Value	Char	LABORATORY EVALUATION CRF
LBSTAT6	Triglycerides ND	Char	LABORATORY EVALUATION CRF

Study D1690R00022 - MEDICAL HISTORY CRF (mh)			
**Variable**	**Label**	**Type**	**Source**
STUDYID	Study Identifier	Char	All CRF
DOMAIN	Domain Abbreviation	Char	All CRF
PTNO	Subject No.	Char	All CRF
VISIT	Visit	Char	All CRF
MHYN1	Cardiovascular disease None	Char	MEDICAL HISTORY CRF
MHTERM1	Cardiovascular disease	Char	MEDICAL HISTORY CRF
MHOTH11	Cardiovascular disease, other specify	Char	MEDICAL HISTORY CRF
MHOTH12	Cardiovascular disease, other specify	Char	MEDICAL HISTORY CRF
MHYN2	Nephropathy None	Char	MEDICAL HISTORY CRF
MHTERM2	Nephropathy	Char	MEDICAL HISTORY CRF
MHOTH2	Nephropathy, other specify	Char	MEDICAL HISTORY CRF
MHYN3	Retinopathy None	Char	MEDICAL HISTORY CRF
MHTERM3	Retinopathy	Char	MEDICAL HISTORY CRF
MHOTH3	Retinopathy, other specify	Char	MEDICAL HISTORY CRF
MHYN4	Neuropathy None	Char	MEDICAL HISTORY CRF
MHTERM4	Neuropathy	Char	MEDICAL HISTORY CRF
MHOTH4	Neuropathy, other specify	Char	MEDICAL HISTORY CRF

Study D1690R00022 - PROTOCOL DEVIATION LIST (pd)			
**Variable**	**Label**	**Type**	**Source**
STUDYID	Study Identifier	Char	All CRF
DOMAIN	Domain Abbreviation	Char	All CRF
PTNO	Subject No.	Char	All CRF
VISIT	Visit	Char	All CRF
SEC	eCRF Section	Char	PROTOCOL DEVIATION LIST
FIE	Field	Char	PROTOCOL DEVIATION LIST
DES1	Description	Char	PROTOCOL DEVIATION LIST
DES2	Description	Char	PROTOCOL DEVIATION LIST
PD	Major PD / Minor PD	Char	PROTOCOL DEVIATION LIST

Study D1690R00022 - PHYSICAL EXAMINATION CRF (pe)			
**Variable**	**Label**	**Type**	**Source**
STUDYID	Study Identifier	Char	All CRF
DOMAIN	Domain Abbreviation	Char	All CRF
PTNO	Subject No.	Char	All CRF
VISIT	Visit	Char	All CRF
PEDAT	Date of Assessment	Num	PHYSICAL EXAMINATION CRF
PESTAT	Not Done	Char	PHYSICAL EXAMINATION CRF
SYSBP	Systolic Blood Pressure	Num	PHYSICAL EXAMINATION CRF
SYSBPND	Systolic Blood Pressure ND	Char	PHYSICAL EXAMINATION CRF
DIABP	Diastolic Blood Pressure	Num	PHYSICAL EXAMINATION CRF
DIABPND	Diastolic Blood Pressure ND	Char	PHYSICAL EXAMINATION CRF
WEIGHT	Weight	Num	PHYSICAL EXAMINATION CRF
WEIGHTND	Weight ND	Char	PHYSICAL EXAMINATION CRF
